# *In Vivo* Application of Tissue-Engineered Veins Using Autologous Peripheral Whole Blood: A Proof of Concept Study

**DOI:** 10.1016/j.ebiom.2014.09.001

**Published:** 2014-09-22

**Authors:** Michael Olausson, Vijay Kumar Kuna, Galyna Travnikova, Henrik Bäckdahl, Pradeep B. Patil, Robert Saalman, Helena Borg, Anders Jeppsson, Suchitra Sumitran-Holgersson

**Affiliations:** a Department of Surgery, University of Gothenburg, Sahlgrenska University Hospital, Gothenburg, Sweden; b SP Technical Research Institute of Sweden, Dept. of Chemistry, Materials and Surfaces, SE-50462 Borås, Sweden; c Department of Paediatrics, University of Gothenburg, Sahlgrenska University Hospital, Gothenburg, Sweden; d Department of Cardiothoracic Surgery, University of Gothenburg, Sahlgrenska University Hospital, Gothenburg, Sweden

**Keywords:** Vein conduits, Vascular diseases, Tissue-engineering, Endothelial precursors

## Abstract

Vascular diseases are increasing health problems affecting > 25 million individuals in westernized societies. Such patients could benefit from transplantation of tissue-engineered vascular grafts using autologous cells. One challenge that has limited this development is the need for cell isolation, and risks associated with *ex vivo* expanded stem cells. Here we demonstrate a novel approach to generate transplantable vascular grafts using decellularized allogeneic vascular scaffolds, repopulated with peripheral whole blood (PWB) *in vitro* in a bioreactor. Circulating, VEGFR-2 +/CD45 + and a smaller fraction of VEGFR-2 +/CD14 + cells contributed to repopulation of the graft. SEM micrographs showed flat cells on the luminal surface of the grafts consistent with endothelial cells. For clinical validation, two autologous PWB tissue-engineered vein conduits were prepared and successfully used for by-pass procedures in two pediatric patients. These results provide a proof of principle for the generation of transplantable vascular grafts using a simple autologous blood sample, making it clinically feasible globally.

## Highlights

• A simple blood sample from the patient can be used to regenerate autologous blood vessels in one week.
• Successful transplantation of two pediatric patients with autologous whole peripheral blood tissue-engineered veins.
• A novel paradigm for treatment, of venous insufficiency, obstructed veins or inadequate autologous veins.

## 1 Introduction

Vascular diseases have been documented to be key components of the global burden of non-communicable diseases. Venous disease of the lower extremities is very common, affecting ∼ 25% of adults in westernized societies (Graham et al., 2003). Peripheral arterial disease is estimated to affect > 25 million patients in Europe and North America alone (Fowkes et al., 1991). These pathological conditions represent an enormous burden, to both patients and healthcare systems. With an aging population, expenditure in the treatment of such diseases is likely to increase significantly over the next 20 years (Raval and Losordo, 2013). Surgical replacement of damaged vessels may include the use of autologous vessels, synthetic grafts such as Dacron or Gore-Tex, or even allogeneic vessels from organ donors. Problems with these alternatives include stenosis, thromboembolization, calcium deposition, infection, the lack of autologous vessels of the proper phenotype or quality, poor patency, donor shortage and the risk of alloimmunization or side effects of immunosuppressive treatment (Veith et al., 1979, O'Donnell et al., 1984, Kurobe et al., 2012). A possible intervention can be replacement with tissue-engineered blood vessels from allogeneic or xenogeneic sources, in which the donor's blood vessel is decellularized (DC) and used as scaffold to repopulate with recipient's autologous stem cells. This approach would make it possible to produce a vessel of the right phenotype and obviate the need for life-long immunosuppression (Olausson et al., 2012).

Studies have demonstrated that unseeded Dacron or Gore-Tex grafts are incorporated in the host blood vessels through a process which is generally referred to as graft healing resulting in endothelialization of the synthetic graft (Pasquinelli et al., 1990). Endothelialization can occur from blood vessel stems at either anastomosis. However, in humans, endothelial cell (EC) ingrowth is very limited, that is, a few millimeters in years (Guidoin et al., 1993). Another mechanism of endothelial healing is, EC coming from circulating cells (Shi et al., 1994, Onuki et al., 1997, Graham et al., 1980, Clowes et al., 1986, Poole-Warren et al., 1996). We and others have shown that circulating ECs, endothelial progenitor cells including the myeloid/monocytic lineages contribute to re-endothelialization (Nowak et al., 2004, Asahara et al., 1997, Bailey et al., 2006). Furthermore, progenitor cells for smooth muscle cells (SMCs) are also present in circulating blood (Simper et al., 2002).

In contrast to experimental animals, the flow surface of synthetic vascular grafts remains unhealed in humans, particularly in the small caliber conduits. Marked differences in graft healing exist between animals and man; and the usual mechanisms of graft endothelialization are partially ineffective in man (Pasquinelli et al., 1990). It has been shown that EC seeding of synthetic grafts prior to implantation has improved the patency of such grafts when compared to unseeded grafts (Wang et al., 1993, Deutsch et al., 2009). Strategies for repopulation of vascular conduits have usually used *in vitro* expanded cells derived from bone-marrow or peripheral blood. Recently, we successfully transplanted a tissue engineered allogeneic vein with *in vitro* expanded autologous bone-marrow derived stem cells. The tissue-engineered graft showed good patency *in vivo*, thus proving a promising and safe clinical approach (Olausson et al., 2012). However, the disadvantage with this approach is that the collection, isolation and large scale expansion of stem cells from tissues or bone-marrow in a limited time with low variability is highly challenging.

All these observations led us to hypothesize that the use of peripheral whole blood (known to contain circulating ECs/endothelial progenitor cells (EPCs)) would result in efficient recellularization (RC) and endothelialization of acellular veins, without the need to isolate and expand subpopulations of angiogenic progenitor cells from bone-marrow or whole blood. Furthermore, an *in vitro* complete and preformed endothelial lining at the time of implantation would help to increase the patency of these engineered vein grafts. Thus, we aimed to develop an easy procedure that would increase the clinical use of the technique and make it globally accessible. We report the clinical transplantation of tissue-engineered allogeneic veins using autologous peripheral whole blood (APWB), in two pediatric patients with portal vein thrombosis.

## 2 Materials and Methods

All research protocols were approved by the Swedish institutional review boards and ethics committees.

### 2.1 Decellularization of Veins

Human iliac or mammary veins about 7–9 cm were retrieved from 14 deceased organ donors after informed consent from the relatives, immediately stored in sterile phosphate buffered saline (PBS) containing 1% penicillin, streptomycin, amphotericin B and transported to the laboratory. On an average, the outer diameter of iliac veins used in the procedures ranged between 1.5 and 2 cm and mammary veins ranged from 0.3–0.7 cm. Decellularization (DC) was carried out as described by us earlier (Olausson et al., 2012) using 1% Triton X-100 and 1% tri-*n*-butyl phosphate (TNBP) and 4 mg/L DNAse under sterile conditions.

### 2.2 Characterization of Decellularized Veins

At the end of the DC process, biopsies were taken from decellularized veins (DV) and processed histologically with Hematoxylin–Eosin (HE), Masson's Trichrome (MT) and Verhoeff Van Gieson (VVG) staining, DNA quantification, Luminex®, scanning and transmission electron microscopic analysis, tensile strength and extracellular matrix (ECM) quantifications. Please see Supplement 1 for further details.

### 2.3 Recellularization of Veins

The entire RC process was performed under sterile conditions and all perfusions were carried out in an incubator at 37 °C supplied with 5% CO_2_. Before RC, the veins were perfused with heparin (Leopharma, Sweden) at a concentration of 50 IU/ml PBS for 2 h. The heparin was drained off and the whole blood was immediately perfused for 48 h at 2 ml/min speed. For collection of blood, see Supplement 1. The blood was then drained off and the veins were washed with PBS containing 1% penicillin–streptomycin–amphotericin for 3–5 min or until the blood was completely removed. The veins were subsequently perfused 4 days with EC and 4 days with SMC media. The complete EC medium contained basal medium MCDB 131, 10% heat inactivated human AB serum, 1% l-glutamine and 1% penicillin–streptomycin–amphotericin and supplemented with EGM-2 Single Quote kit (Lonza, Walkersville, MD USA). A commercially available SMC medium (Cascade Biologics) containing growth and differentiation factor supplements was used. The veins were recellularized for a total of 10 days.

### 2.4 Characterization of Recellularized Veins

To visualize the presence of EC, antibodies to CD31 (1:1000) (Abcam, Germany) and von Willebrand factor (1:100) (Santa Cruz, Germany) were selected and stained by immunofluorescence, while smooth muscle actin (1:50) (Abcam, Germany) to visualize SMC. The tensile strength of recellularized veins (n = 3), work and elongation was compared to normal and decellularized veins.

### 2.5 Enumeration of Cells in the Repopulated Veins

Quantification of cell numbers in the lumen was done by staining for the endothelial cell marker VEGFR-2 and monocyte marker CD14. Single and double-positive cells were quantified manually. On average, 15 images of the lumen were taken per vein covering an area of 47 500 μm^2^. The data is represented as the number of cells per square centimeter area of the vein and the values are represented with standard error mean of the original value.

### 2.6 Clinical Transplantations

#### 2.6.1 Patient 1

A 4-year-old girl was referred to the children's hospital in Gothenburg after a history of fatigue, a slightly elevated body temperature and a tender tumor in the left upper abdominal quadrant present since 1 month of age. Initial tests at 4 years showed anemia, thrombocytopenia, and neutropenia. A CT-angiography showed no portal circulation from the superior mesenteric vein to the liver (Fig. 4F) and problems in detecting the intrahepatic portal vascular system. The umbilical vein was not open and the jugular veins were estimated to be too short as the only source of vein graft (also see surgical procedure and post-operative monitoring in the Supplement methods section). Splenomegaly and cavernous vein formation was found in the hilum. An endoscopy demonstrated esophagus varices from 15 to 25 cm down to the cardiac region. The child was evaluated for a possible Mesorex procedure by a pediatric multidisciplinary team. The child weighed 17 kg and was 107 cm in height.

#### 2.6.2 Patient 2

A 24-month-old girl, weighing 7.4 kg, was referred to the children's hospital with a history of upper abdominal pain coinciding with feeding of the child, starting at 8 months after birth. Initial investigation at a local hospital found splenomegaly and pancytopenia. A hematological work up was done and found normal. A CT-angiography revealed the absence of the portal vein and difficulties with detection of any intrahepatic portal vascular system (Fig. 4G). No patent umbilical vein could be detected. Endoscopy showed prominent varices explaining the pain correlating to food intake. The child was evaluated for a possible Mesorex procedure by a pediatric multidisciplinary team. The child weighed 7.4 kg and was 73.5 cm in height.

Since no suitable autologous veins were detected in both the patients, two decellularized veins were recellularized under good management practice (GMP) facilities available at The Sahlgrenska University Hospital-Gothenburg-Sweden as described above with 25 ml autologous blood taken from the two patients and used for the Mesorex procedures.

#### 2.6.3 The Ethical Process

As in the previous published case (Poole-Warren et al., 1996) in the present two cases, a pediatric team performed the evaluation in a multidisciplinary fashion, which also includes abiding to the UNICEF Convention on the Rights of the Child. The parents were informed several times and given details of the procedure, the possible anticipated complications and the alternative remaining with a failed surgical correction of the EHPVO. This information was given to the parents by the responsible surgeon together with members of the pediatric team. The parents also provided informed consent, which was given to the pediatric team in the absence of the operating surgeon to avoid pressure. Moreover, permissions from the two regulatory bodies in Sweden—The National Medical Board and The Medical Products Agency were granted. Permission from members of The Local Ethical Board (Etisk Forum) was also obtained prior to proceeding with the transplantation.

Please see Supplement 1 for post-transplant monitoring and statistics.

## 3 Results

### 3.1 Decellularized Veins and Extracellular Matrix

**Fig. 1:**
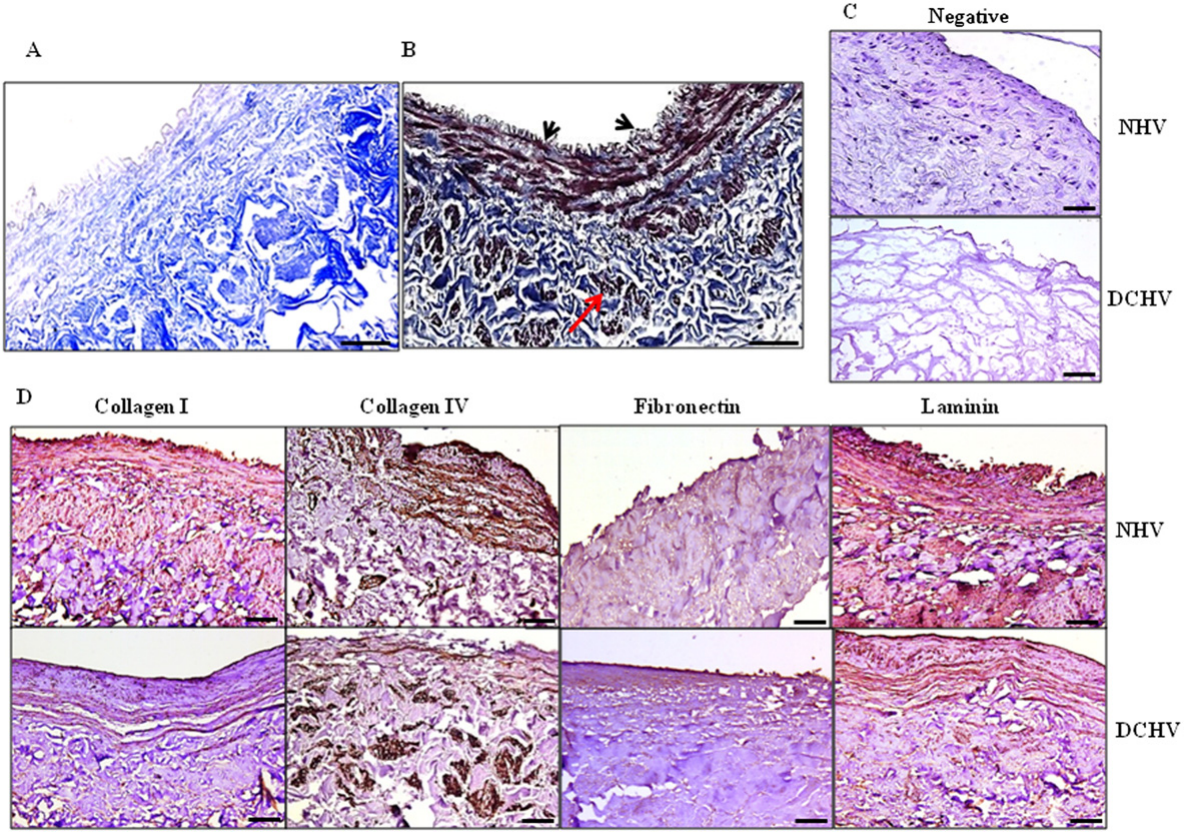
A representative microscopic view of decellularized vein grafts. Masson's Trichrome staining of (A) decellularized vein (DV) showing no nuclei, indicating lack of endothelial and smooth muscle cells, but with abundant collagen still present (blue) and (B) normal vein showing presence of nuclei (black, arrows), cytoplasm (red/pink) and collagen (blue). (C) Negative controls. (D) Immunohistochemical staining of normal and DV for the various extracellular matrix components. Nuclei are seen only in normal but not in DV. In the DV although no cells/nuclei are seen, the ECM components stained positive indicating the retainment of important ECM proteins (n = 14). A–D: Scale bar = 75 μm.

Nine cycles of decellularization with 1% Triton and 1% tri-*n*-butyl phosphate resulted in generation of pale and translucent veins (Supplement Fig. S1A, DV). The veins were successfully decellularized in 12 days as evidenced by lack of nuclei when compared to normals (Fig. 1A–C, also see Supplement Fig. S1B–E) and confirmed by electron microscopy (Supplement Fig. S1F–H). Major ECM proteins such as collagen I, collagen IV, fibronectin and laminin (Fig. 1D) were preserved in DV. The quantity of GAGs (p = 0.041) but not collagen (p = 0.700) was significantly decreased in veins after DC (Supplement Fig. S2A). A significant decrease in DNA concentration was found in DV (15.5 ± 7.93 ng/mg) as compared to 244.5 ± 65.01 ng/mg of tissue in normal veins (p = 0.007). Several growth factors albeit lower were still present in the DV (Supplement Fig. S2B). Tensile strength testing of the DC veins showed a decrease in the work needed to deform the samples completely when compared to normal samples and the elongation measured at 50% of total work had significantly decreased after DC (p < 0.05, Supplement Fig. S2C & D).

### 3.2 Recellularization of Veins

**Fig. 2:**
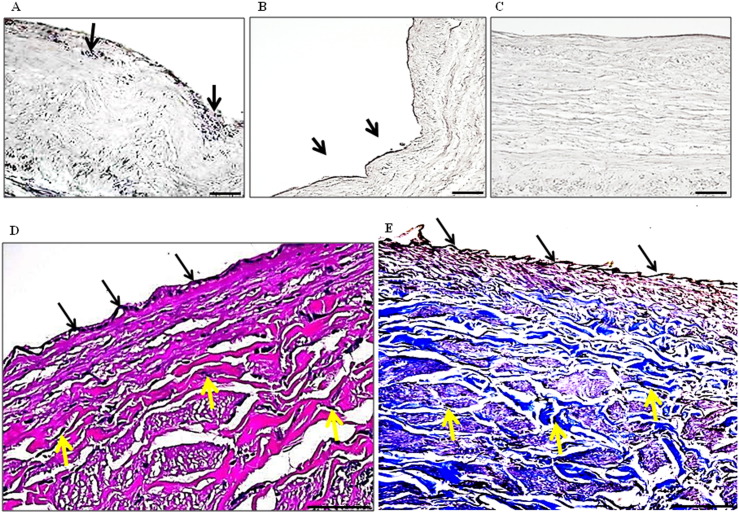
Microscopic view of a representative bioengineered vein graft with peripheral whole blood. (A) Picture shows migration of cells (black) into the tissue after perfusion with blood, (B & C) presence of an endothelial cell monolayer on the luminal side after perfusion with endothelial cell medium and an overall improved architecture of the vein. Staining of tissue-engineered human veins recellularized with peripheral whole blood by (D) Verhoeff Van Gieson, showed presence of nuclei (black) for endothelial (black arrows) and smooth muscle cells (yellow arrows). The cytoplasm was stained pink and elastin black. (E) Masson's Trichrome staining showed presence of black nuclei (black), blue collagen and pink cytoplasm (n = 5). A–C: Scale bar = 75 μm, D & E: scale bar = 25 μm.

The gross morphology of veins after RC looked pinkish with good tunica externa (Supplement Fig. S1A, RV). Histological examination two days after perfusion of DV with whole peripheral blood showed migration of cells into the tissue, (Fig. 2A). Four-day perfusion with EC medium resulted in an almost confluent monolayer of cells on the lumen of the vein (Fig. 2B) and immigrated cells in the media (Fig. 2C). This is evidenced by single immunofluorescent staining for CD31 (Supplement Fig. S3E) and alpha smooth muscle actin (Supplement Fig. S3D) staining. VVG and MT staining of the veins also confirmed the presence of nuclei (black), cytoplasm (red in MT) and collagen (red in VVG and blue in MT) (Fig. 2D & E respectively).

**Fig. 3:**
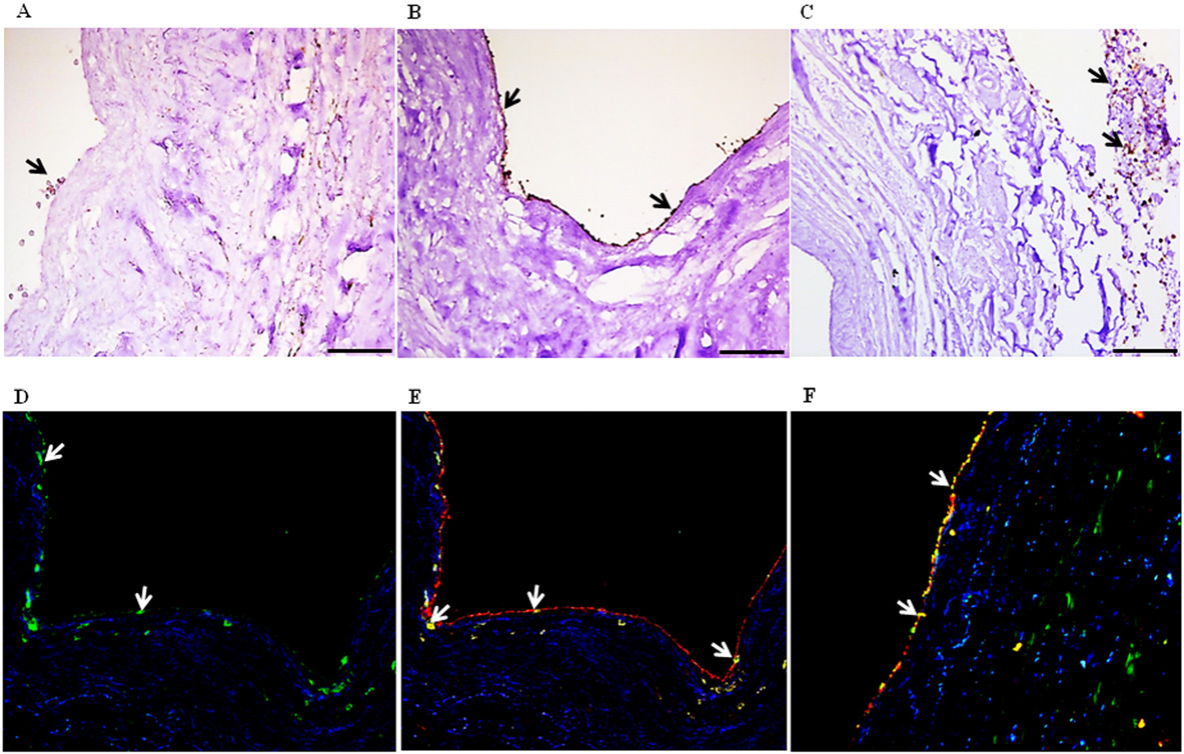
Phenotypic labeling of peripheral whole blood cells involved in repopulation of the bioengineered veins. (A) Cells staining positive (brown) for the tyrosine kinase receptor VEGFR-2 are found adhering to the lumen of the scaffold after perfusion of peripheral blood for 48 h. (B) These VEGFR-2 + cells formed a monolayer on the luminal side of the bioengineered vein after perfusion of endothelial cell medium for 4 days (brown). (C) Cells expressing the common leucocyte marker CD45 were found mainly in the media and adventitia (arrows). (D & E) A small fraction of cells in the lumen also stain positive for the monocyte marker CD14 (green), while double staining showed that some were also double positive (arrows) for VEGFR-2 (red). (F) Double staining showing that VEGFR-2 + cells were also positive for the endothelial marker CD31. Yellow color indicates co-localization of the two markers. The repopulating cells were negative for other markers such as CD3, CD19, CD68, CD133, CD34, CD56 and CD61 (n = 6).

**Fig. 4:**
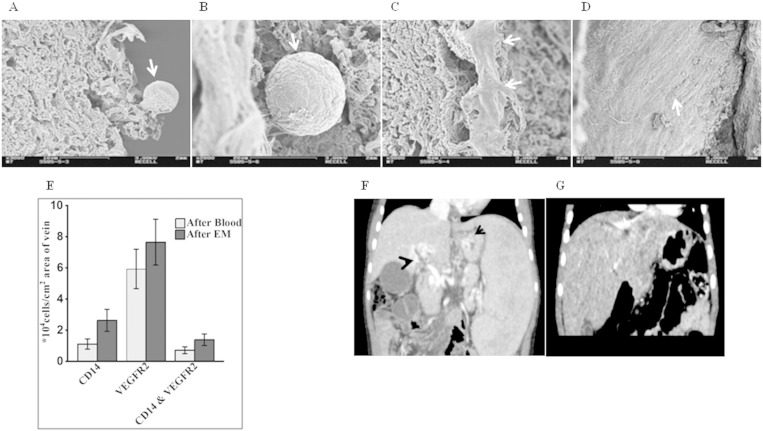
Characterization of the recellularized veins and pre-transplant clinical data of the two patients. (A & B) Scanning electron micrographs of the recellularized veins showing binding of cells (arrows) to the lumen and in the media after perfusion with peripheral whole blood. (C & D) Further perfusion with endothelial cells medium resulted in formation of a continous smooth endothelial cell layer (arrows). (E) Higher numbers of VEGFR-2 + cells were found in the recellularized veins (n = 6) as compared to CD14 + cells both after perfusion with blood and endothelial cell medium (EM). (F & G) CT-angiographs of patients 1 & 2 respectively before transplantation. (F) Patient 1: showing varicose veins in the hilum (blue) and at the esophagus (black). (G) Patient 2: showing no intrahepatic portal system. In both cases, no portal circulation is observed.

Phenotypic characterization of the peripheral blood cells involved in formation of neo-endothelialization demonstrated that many cells expressed the tyrosine kinase receptor-vascular endothelial growth factor receptor-2 (VEGFR-2) (Fig. 3A; after blood & B; after endothelial cell medium). Negative controls are shown in Supplement Fig. S3A–C. Cells positive for the leukocyte common antigen CD45 were found mainly in the adventitia and not the lumen of the veins (Fig. 3C), indicating that these cells mainly differentiated to smooth muscle cells as evidenced by positive staining for alpha smooth muscle cell actin. A smaller fraction of CD14 + cells (green, Fig. 3D) which also stained positive for VEGFR-2 (red, yellow indicates co-localization; arrows) contributed to this process (Fig. 3E). A continuous monolayer of endothelial cells staining positive for VEGFR-2 and the EC marker CD31 (arrows) was observed after 4 days with EC medium (Fig. 3F). These findings were corroborated by scanning electron microscopy (Fig. 4A–C) which also revealed the presence of flattened cells along the luminal surface of the veins consistent with endothelial cells (Fig. 4D). The cells were negative for the markers CD3, CD19, CD68, CD133, CD34, CD56 and CD61, indicating that the major cell types contributing to repopulation are VEGFR-2 +/CD45 + and VEGFR-2 +/CD14 + cells.

Our initial results showed no difference in the numbers of cells at 6 days and 10 days, therefore enumeration data is presented at two time points *i.e.* immediately after perfusion with blood (48 h) and after perfusion with EC medium for four days (total 6 days). On an average, we found 11.5 ± 0.02 × 10^4^ DAPI + cells/cm^2^ in the lumen of normal control veins. In the recellularized veins, staining for VEGFR-2 after perfusion with whole blood demonstrated an average of 5.93 ± 1.26 × 10^4^ cells/cm^2^. The numbers of VEGFR-2 + cells were increased after perfusion with EC medium (7.65 ± 1.47 × 10^4^ cells/cm^2^). This was however not statistically significant (p = 0.387). In addition, the number of CD14 + cells after two days of perfusion with blood showed an average of 1.11 ± 0.32 × 10^4^ cells/cm^2^, and the numbers of these cells were also not significantly increased after perfusion with EC medium (2.63 ± 0.70 × 10^4^ cells/cm^2^, p = 0.132). Double staining of the cells in the lumen for CD14 and VEGFR-2 revealed that approximately half of the CD14 + cells also expressed VEGFR-2 (Fig. 4E) (0.71 ± 0.22 × 10^4^ cells/cm^2^ with blood and 1.39 ± 0.37 × 10^4^ cells/cm^2^ after EC medium).

Sterility testing showed no marked increase in absorbance during 14 days of media culture. The average of optical densities at 600 nm measured in spectrophotometer for fresh media (0.006) was similar to that of perfused media (0.009) and not significantly different (p = 0.251). The average values for negative control and positive control are 0.010 and 0.36 respectively. No microbial colonies were seen on the agar plates. Biomechanical analyses showed that the work increased significantly after RC while no significant changes were seen in the elongation between RC and DC samples (Fig. S2C & D). No significant difference was found between normal and RC samples, indicating that the biomechanical properties of the RC veins are similar to the native veins (Supplement Fig. S2C & D).

### 3.3 Clinical Cases

To test the *in vivo* patency of these tissue-engineered veins, two pediatric patients suffering with extra hepatic portal vein obstruction (EHPVO) were transplanted with tissue engineered veins regenerated using 25 ml autologous peripheral blood on compassionate basis. Fig. 4F shows a CT-angiography demonstrating no portal circulation from the superior mesenteric vein to the liver and problems in detecting the intrahepatic portal vascular system in patient 1. Similarly, Fig. 4G shows a CT-angiography demonstrating the absence of the portal vein and difficulties with detection of any intrahepatic portal vascular system in patient 2.

### 3.4 Clinical Transplantation Data

**Fig. 5:**
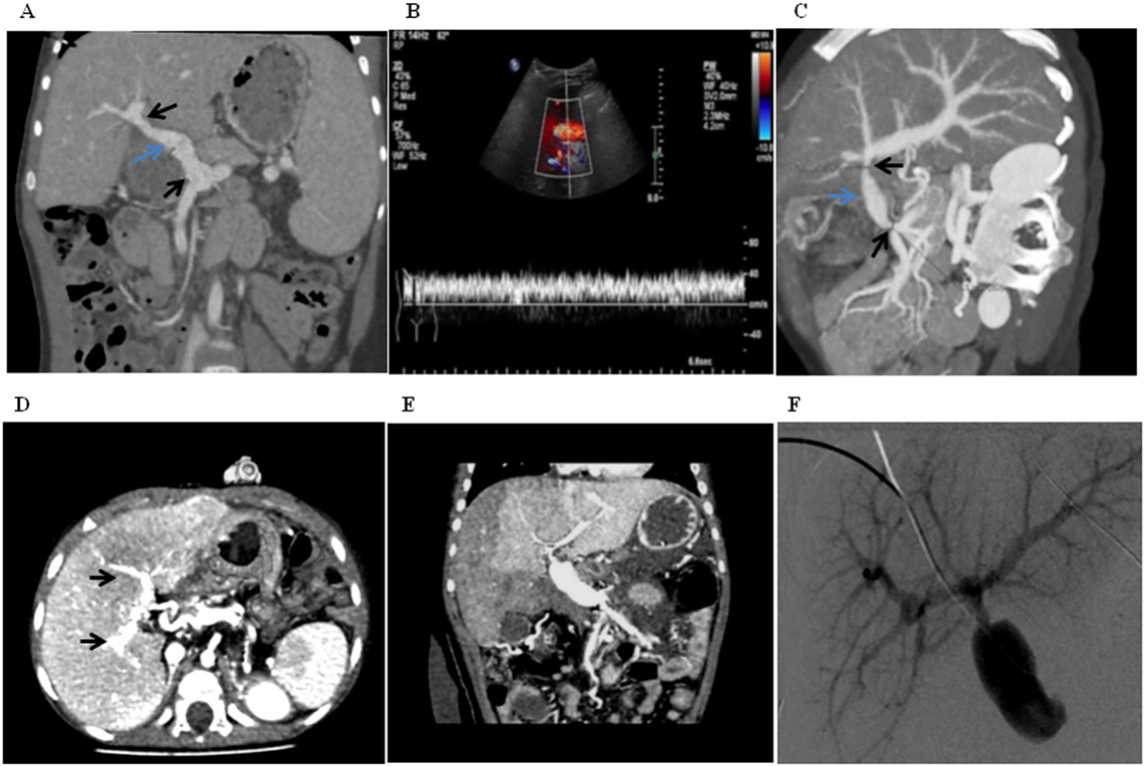
Post-transplant clinical data for the two transplanted patients. (A) Patient 1: Postoperative CT-angiograph, showing the new graft (blue arrow) with good anastomotic sites (black arrows) and well established intrahepatic portal system. (B) The postoperative period was uneventful with good intrahepatic blood flows. The flow velocity showed 30–40 cm/s both in the portal vein and in the hepatic artery. (C) Patient 2: CT-angiography after first transplantation, showing a revascularized portal flow and intrahepatic portal system (blue arrow). At the six-month checkup, the patient had a reduced diameter at both anastomotic sites, (black arrows), however the intrahepatic portal vein system was significantly developed (D). Seven months later the patient received a second graft. (E) CT angiography day after surgery of the second graft revealed poor perfusion of the right portal system. (F) However, after a percutaneous angiography the right portal system was well perfused.

Patient 1 received an 8 cm long vein, anastomosed to the superior mesenteric vein and the left portal vein as earlier described (Fig. 5A) Olausson et al., 2012. The central venous pressure (CVP) was 11 cm H_2_O and the portal pressure dropped from 22 cm to 14 cm after reperfusion of the vein — a difference of 3 cm compared to the pressure in the right atrium. The flow velocity showed 30–40 cm/s both in the portal vein and hepatic artery (Fig. 5B). Twenty-one months post-transplantation the patient has an unchanged image as seen in a 3D reconstruction of the graft (see Video in Supplement). The esophageal varices have diminished (endoscopy) and clinical laboratory parameters are normal. No surgical or other complications have been observed during frequent regular controls and ultrasounds.

Patient 2 received a 6–7 cm long vein, initially anastomosed to the patient's own 5 cm long umbilical vein. This had to be adjusted perioperatively due to thrombosis in the umbilical vein section, resulting in an anastomosis between the vein graft and the left portal vein as earlier described (Olausson et al., 2012). The gradient between the cava and the intrahepatic system was < 1 cm H_2_O, indicating good liver parenchyma, whereas the gradient between the portal vein and the mesenteric system remained 6 cm, indicating a partial stricture. On the second day after surgery the patient was revised due to a narrowing at the site of anastomosis in the liver (Fig. 5C). After revision, both anastomoses were patent, but difficulties to lower the difference between the CVP and the mesenteric pressure below 8 cm H_2_O were encountered. The postoperative period was otherwise uneventful with good intrahepatic blood flows. At the 6-month checkup, the patient had a reduced diameter at both anastomotic sites, however the intrahepatic portal vein system was significantly developed (Fig. 5D). An attempt to dilate the narrow anastomosis on the mesenteric side resulted in thrombosis of the graft. Seven months after the first procedure a decision to revise the graft was taken and the patient received a second vein, prepared exactly as the first. During this procedure difficulties to open up the right portal vascular bed was evident. The right portal system was difficult to reach with a surgical microprobe. After reperfusion a remaining gradient of 10 cm H_2_O was detected. CT angiography the day after the surgery revealed poor perfusion of the right portal system (Fig. 5E). Four days later a percutaneous angiography was performed with measurements of the gradient over the anastomoses. The right portal system was now well perfused and the gradient was only 3 cm H_2_O (Fig. 5F). Further controls have been uneventful, (Nineteen months after the procedure). Both patients received heparin infusions for 2 weeks followed by warfarin for 1 year as anti-coagulation therapy.

For both patients INR improved from (1.6–1.8) prior to surgery to (1.0–1.2) 2 weeks after surgery. This was similar to the experience previously reported in the first patient. Platelet count as well as hemoglobin and leucocytes also improved. At 1.5 years post-implantation, both patients are less tired and symptoms related to food intake for patient 2 disappeared after surgery. Furthermore, no antibodies to HLA class I or II antigens were found. Patient 1 now weighs 20.3 kg and is 118.7 cm long, while patient 2 weighs 10 kg and is 87 cm long.

## 4 Discussion

Our study is a proof-of-concept clinical report of the successful recellularization of decellularized human blood vessels with autologous peripheral whole blood, which were subsequently used for a bypass procedure in two patients with portal vein thrombosis without the need for immunosuppression. The work is important conceptually because it provides early evidence for generating clinically useful personalized blood vessels using a simple blood sample from the patient. The work also establishes the feasibility and safety of a novel paradigm for treatment, in cases of venous insufficiency, obstructed veins or inadequate autologous veins within a short period of time. This method of recellularization is an easy procedure that will increase the clinical application of the technique and make it globally feasible.

Recellularization using blood is advantageous over the use of *in vitro* expanded autologous stem cells as one may reduce the risk of spontaneous mutations that may be associated with expanded cells. One clinical study (Hibino et al., 2010) reports successful outcomes in their thoracic venous conduit using a bio-absorbable graft incubated for 2 h with an inoculum of bone marrow prior to implantation without the need for extended *ex vivo* culture. However, aspiration of bone-marrow is an invasive technique, needing anesthesia and specialized personnel to perform the procedure and may also be associated with risk for infection. The short incubation time with isolated cells will not permit the formation of an EC layer, which is a crucial factor for clinical patency, and function of the blood vessel. Our approach promises a simplified convenient technique. We speculate that the ECM proteins and growth factors retained in the tissues after DC may enhance the attachment and growth of the recipient cells in the tissue (Li et al., 2004). At present, the definition of EPC remains controversial and is not yet consistent (Yoder and Ingram, 2009). Most commonly, marker combinations for identifying the putative circulating EPC comprise certain hematopoietic lineage markers, such as, CD133, CD34, VEGFR-2, Tie-2, and UEA-1 lectin (Yoder and Ingram, 2009, Peichev et al., 2000) and circulating myeloid cells expressing CD14 (Hristov et al., 2007, Venneri et al., 2007) — corresponding to a heterogeneous cell population possessing an overlapping phenotype with endothelial cells and hematopoietic progenitors. PBMC are also capable of developing into fibrocytes — potential vascular progenitors (Bucala et al., 1994). Fibrocytes express the common leukocyte antigen CD45 and are variably reported to express CD14 and CD34 (Medbury et al., 2008). These cells are also known to express collagen I and the alpha-smooth muscle actin. In the present study, phenotypic characterization of the cells from PWB repopulating the veins demonstrated that the majority of the cells expressed VEGFR-2, while a small fraction was also double positive for the monocyte marker CD14 (VEGFR-2 +/CD14 +), indicating that the major cell types are derived from the monocytic/macrophage lineage, confirming results from several published reports (Nowak et al., 2004, Asahara et al., 1997, Bailey et al., 2006, Simper et al., 2002). Interestingly, cells that migrated into the media were found to be CD45 + which also stained positive for the marker smooth muscle cell alpha actin, indicating that this cell population may include precursors of smooth muscle cells. Thus, regeneration of blood vessels can attract a variety of endothelial and smooth muscle cell precursors that may differentially express the VEGFR-2, CD45 and CD14 receptors. Perfusion with endothelial cell medium, increased, although not significantly, the numbers of VEGFR-2 + cells, indicating proliferation of these cells. We also observed the formation of a complete EC monolayer. Thus, perfusion with PWB is the most important step for endothelialization, since perfusion with PWB will permit binding of many endothelial precursor cells, while perfusion with EC medium (which contains VEGF and other growth factors) may help in proliferation and formation of an EC monolayer.

However, perfusion with smooth muscle cell medium did not significantly affect any of these parameters. As a consequence, we have decided to skip perfusion with SMC medium.

We have reported earlier that peripheral blood cells expressing the tyrosine kinase receptors VEGFR-2 or Tie-2 define functionally competent cell populations capable of re-endothelialization and differentiation into smooth muscle cells, the numbers of which are ≈ 0.8% and 2% respectively in PWB (Nowak et al., 2004). In the present study enumeration of VEGFR-2 + cells after perfusion with 25 ml blood from various donors showed an average cell count of 7.65 ± 1.47 × 10^4^ cells/cm^2^ in RV which was similar to the numbers reported by Ranjan AK et al. who used 6.6 × 10^4^ EPC for endothelialization of small diameter vascular prosthesis (Ranjan et al., 2009). The volume of 25 ml blood for recellularization was chosen for the following two reasons, a) this was the minimum amount required for perfusion through the vein and the bioreactor system and b) consideration for pediatric patients with fragile blood vessels. Larger volumes of blood did not result in reduced time needed for repopulation (unpublished observations), although this finding needs to be verified in a larger study.

In the present and other currently ongoing studies we have successfully recellularized veins using blood from individuals and patients in the age range of 4–55 years. However, it is reported that the numbers of circulating stem/precursor cells is decreased in patients with diabetes (van Ark et al., 2012) and end-stage renal diseases (Westerweel et al., 2007). So it remains to be tested whether this method would work in such patients. We did not detect any HLA antibodies after transplantation indicating satisfactory decellularization of the blood vessels. The patients have been followed for one year and Nine months and one year and Seven months respectively. Although patient 2 had to be re-operated and problems with the anastomoses could be noted, this was evident already at the primary operation. Small children or children with inferior intrahepatic portal vein system may require sequential surgery. The second intervention however, opened up of the obstructed portal bed and diminished the initial gradient over the anastomosis. Taking into account that the alternative treatment for these patients is liver transplantation including lifelong immunosuppression, a revision must be seen as a minor drawback.

Both patients have been transplanted on compassionate grounds and therefore optimization of the technique has been on a “patient to patient” basis. We are currently seeking permits to carry out a clinical trial, which will include a larger number of patients to determine the efficacy of grafting tissue-engineered veins as vascular replacement therapy.

The possibility of using peripheral blood to regenerate a decellularized vessel is a major step towards making tissue-engineered blood vessels a feasible technique globally for clinical application and our results prove the clinical potential of this method in the treatment of patients with vascular diseases.

In conclusion, the possibility of using peripheral blood to regenerate a decellularized vessel is a major step towards making tissue-engineered blood vessels a feasible technique globally for clinical application and our results prove the clinical potential of this method in the treatment of patients with vascular diseases.

The following are the supplementary data related to this article.

Supplementary material. 3D reconstruction of vein graft between superior mesenteric vein and left intrahepatic portal vein.

Video 3-D reconstructed graft.
Demonstration of a good vascular bed in patient 1, 21 months post-transplantation.

## Conflicts of Interest

SSH is a cofounder and a board member of NovaHep AB, a company that has licensed the technology of tissue-engineering blood vessels. SSH also receives royalties from Absorber AB, and has a pending patent for tissue-engineering of veins.

MO has a pending patent for tissue-engineering of veins; licensed to NovaHep AB and for which he will receive a royalty should the invention reach the market.

HBä reports grants from Laboratory for Transplantation and Regenerative Medicine, during the conduct of the study.

The other authors declared no conflicts of interest.

## Author Contributions

SSH conceived, designed and oversaw all of the *in vitro* studies, collection of results, interpretation of the data, and writing of the manuscript. SSH is also responsible for the new recellularization protocol. MO is the senior surgeon who did the surgery and perioperative care. MO was also responsible for collection, interpretation and writing of the clinical data. VKK did the all preclinical work, and supervised the whole procedure and wrote the report. GT assisted in the development of the decellularization process for matrix creation, the seeding procedure, and did immunofluorescence histology. PBP designed the bioreactor. HBä designed and performed all the biomechanical studies and co-wrote the paper. RS and HBo performed patient screening and follow-up. AJ supplied blood vessels for the study and co-wrote the paper. All authors have seen and approved the final version of the report to be published.

**Funding**: Swedish Government. The sponsor of the study has no role in the study design, data collection, data analysis, data interpretation, or writing of the report.
