# Supplementary material for: In Vivo Application of Tissue-Engineered Veins Using Autologous Peripheral Whole Blood: A Proof of Concept Study
Source: eBioMedicine. 2014 Sep 22;1(1):72–9. doi: 10.1016/j.ebiom.2014.09.001 (PMC4457407; doi:10.1016/j.ebiom.2014.09.001)
Supplement: Supplementary file 1 — Supplementary material. 3D reconstruction of vein graft between superior mesenteric vein and left intrahepatic portal vein. [file mmc1.pdf]

Online Methods

Histology, immunohistochemistry and immunofluorescence

Normal (n=10), decellularized (n=14) and recellularized (n=14) vein biopsies were processed according to standard procedure. ECM proteins were detected by immunohistochemistry The primary antibody concentrations used were collagen I (1:100) (bs-7158R, Bioss, USA), collagen IV (1:100) (bs-0806R, Bioss, USA), fibronectin (1:500) (ab23751, Abcam, Germany) and laminin (1:100) (bs8561R, Bioss , USA).

Biopsies were either fixed in 4% buffered formalin for 48h or directly frozen at -80^{0}C in OCT to prepare to prepare paraffin and frozen sections respectively. Five µm thickness sections were cut for staining. The paraffin sections after rehydration in descending series of alcohols were stained with Hematoxilin-Eosin (HE), Massons Trichrome (MT), Vernhoeff Von Gieson (VVG) stains and with various antibodies for immunohistochemistry. In HE staining the slides were incubated in Meyers Hematoxylin and alcoholic eosin for 7 and 1 min respectively, followed by washing with distilled water in-between for 10min, dehydrated and mounted. The MT (25088-1, Polysciences, Germany) and VVG (25089-1, Polysciences, Germany) stainings were performed according to the manufacturer's instructions. For immunohistochemistry, the sections were dehydrated, antigen retrieved in 10mM boiling sodium citrate buffer (pH 6) for 20min, blocked for 40min (X0909, Dako, USA) and incubated with primary antibody overnight at 4^{0}C. The slides were washed with PBS and incubated for 10min with secondary antibody (878963, Invitrogen) and identified with DAB (K3468, Dako, USA). For immunofluorescence, the protocol followed was the same until the antigen retrieval step, and then blocked with 5% serum for 30 min, incubated with primary antibody overnight at 4^{0}C, washed and incubated with Alexa 488 or 568 secondary antibodies (A11001, A21202, A11057, A11011 Life Technologies, Sweden) for 40 min, counter-stained

with 4´, 6-diamidino-2-phenylindole (DAPI) for 30seconds and mounted. Primary antibodies used were anti-CD3 (M7254292, Dako, USA), -CD19 (M7296292, Dako, USA), -CD34 (M7165292, Dako, USA), -CD45 (M0701292, Dako, USA), -CD56 (M7304292, Dako, USA), -CD61 (M0753, Dako, USA), -CD68 (M0876292, Dako, USA), -CD14 (ab45870, Abcam, Germany), -CD31 (ab9498, abcam, Germany), -CD133 (130090422, Miltenyi Biotec, Germany) and –VEGFR-2 (AM21042PU-S, Acris Antibodies, USA). Immunofluorescence staining was performed to detect double-positive CD14+/CD31+, CD31+/ VEGFR-2+, CD14+/VEGFR-2+ and CD45+/VEGFR-2+ cells.

Collagen and glycosaminoglycan quantification

Collagen and glycosaminoglycans (GAGs) quantification was done using the Sircol Collagen Assay kit and Blyscan GAGs Assay kit from Biocolor Company as described by us earlier[40].

DNA quantification

Twenty mgs of tissue was collected from five normal and 14 decellularized veins and the DNA isolated using DNeasy blood and tissue kit protocol (Qiagen, Sweden). Amount of DNA present was measured with nanodrop (ND-1000, Saveen Werner, USA).

Angiogenic growth factor quantification by Luminex

Thirty mgs of tissue sample was taken from two different veins before and after decellularization and total protein was isolated by digesting mechanically for 4 min in tissue rotor at high speed using a commercially available kit (2140, Millipore, Germany). The amount of protein was measured by the standard Bradford method and measured at 595nm using an ELISA reader (Synergy2, Biotek, USA). The protein amount of all tissues was normalized to same concentration with TM buffer (Millipore, Germany) and loaded onto

Luminex plate. A commercially available kit from Millipore (Milliplex MAP Human Angiogenesis/Growth Factor Magnetic Bead Panel, HAGP1MAG-12K) which included 17 analytes was used to detect angiogenic growth factors in normal and decellularized vein tissues using the Luminex technology. Luminex® was performed according to the human angiogenesis/growth factor magnetic bead panel 1 supplier´s protocol (Millipore, Sweden).

Electron microscopy

Pieces from decellularized and recellularized veins (n=3) were cut with razor blades at sizes suitable for scanning electron microscopy (SEM) and analyzed at the EM core facility at University of Gothenburg. Specimens were fixed in a mixture of 2%, paraformaldehyde and 2.5% glutaraldehyde in 0.05 M sodium cacodylate buffer, pH 7.2. SEM objects were subjected to repeated treatment with osmium tetroxide[41] followed by dehydration in ethanol and hexamethydisilazane, which was allowed to evaporate. The dried tissue blocks were mounted on aluminum stubs and sputter coated with palladium before examination in a Zeiss 982 Gemini scanning electron microscope.

Collection of blood

On the day of recellularization (RC), 25 ml blood was collected from each healthy donor (age group 25-35) in sterile heparin coated Vacutainer™ tubes and transported to laboratory as soon as possible (within 1-2 hours). The volume of blood required depends on length of vessel and length of pipes used in the bioreactor. A vein of 9 cm length and 1cm in diameter can be recellularized with 25 ml blood.

Bioreactor

A bioreactor was prepared indigenously in the laboratory depending on the dimensions of the veins. The bioreactor assembly consists of culture chamber, silicon circuit (5mm inner diameter) and peristaltic pump. The culture chamber that holds the vein scaffold is a falcon tube made of polypropylene-polyethylene and via silicon tubes blood and media were continuously perfused at 2ml/min speed using a peristaltic pump. The complete bioreactor assembly was sterilized in an autoclave prior to use. The culture chamber is about 20cm with an inlet and outlet. Both the ends of the vein are fixed to the connectors with silk sutures and placed vertically in the culture chamber. The 5mm diameter silicon tubes connect chamber inlet and outlet. Since the media flows through the vein from bottom to top and the peristaltic pump continuously perfuses the medium, the vein remains inflated and the entire surface is exposed to nutrient media. (Supplement Fig. S4).

Sterility Control Test

To evaluate the sterility of vein during recellularization, the endothelial and smooth muscle cell media before and after perfusion were collected and tested for microbial growth.
Two growth media Fluid Thioglycollate broth and Tryptone soya agar that support the growth of broad range of microorganisms (fungi, aerobic & anaerobic bacteria) were selected and 500µl of collected media were added onto agar plate and into broth and kept at 37^0C for 14 days. Tryptone soya agar plate and broth alone were used as negative controls while broth and agar surface contaminated by local exposure was used as positive control. During incubation, after every 2 days, 200µl of broth was collected and checked for absorbance at 600nm in spectrophotometer and the differences in optical density were recorded. The agar plates were observed for presence of microbial colonies after 14 days.

Tensile strength measurement

Vein segments were tensile tested with an Instron 5566 (Instron, Norwood USA). The pre-load was 0.1N and the test speed was 50mm/minute. The accuracy of the tensile tester was 0.5% in force and 0.5% in elongation. The vein was cut into approximately 4mm wide ring shaped samples. The smallest width of the sections was measured with a caliper and recorded. Two cylindrical 5mm grips (each 2.5mm high and 5mm wide) were placed inside the ring samples and the elongation of the samples was measured after pre-load (Supplement Fig. S2B). Since the shape of the resulting tensile test diagrams varied, work and elongation at 50% total work was calculated and used for comparing normal (n=4), decellualrized (n=10) and recellularized (n=3) samples. The work was normalized by dividing the measured force with the smallest width of the rings; since this is the part experiencing the load (stress was not calculated since the blood vessel wall was not homogenous).

Surgical procedure and post-operative monitoring

Both patients had a preoperative CT angiography to plan for possible accessible autologous veins on the neck for the Mesorex procedure. In both cases, the initial decision was to explore the umbilical vein and the superior mesenteric vein. If both these sites were adjudged surgically fit, the umbilical or other abdominal vein would be used as first choice for the bypass. A suitable jugular vein was considered as second choice. However, in both cases suitable autologous veins were not found.

Both patients were operated using the same technique as previously described[28]. In Patient 1, an umbilical vein was present but was not suitable for use. The site for the anastomoses was at the junction of the umbilical vein in the left portal vein. An eight cm vein graft was used. Patient 2 had an umbilical vein that initially was used for the anastomosis of the vein graft. The central venous pressure (CVP) was calibrated (cm H_2O) and measurement of the portal

pressure before and after reperfusion of the vessel was performed and related to the CVP. Pre- and postoperative ultrasounds with estimates of flow velocities (cm/s) were performed twice daily first week and once daily the second week. Screening for antibodies to Major histocompatibility complex antigens was performed using the technology of luminex.

Screening for antibodies to Major histocompatibility complex antigens

We screened for HLA class I and II antibodies both pre and post transplantation. Serum samples were collected one month prior to transplantation, 1, 4weeks, and 9 months post-transplantation for patient one, while for patient two, samples were collected at 2 weeks and 6 months post-tx. Presence of HLA antibodies was detected by standard Luminex procedure using LABScreen™ Mixed assay (One Lambda Inc, CA, USA).

Statistics

Mann-Whitney U test were performed to compare the effects of DC and RC on vein samples in all experiments. A p value < 0.05 was considered statistically significant. Statistical analysis was performed using Graph pad prism version 4.0. All values represent average of that experiment and for drawing graphs all values of same group were calculated mean±s.e.m.

Supplement Figure Legends

Figure S1: (**A**) Gross morphology of decellularized veins (DV) showing a pale and translucent scaffold. (**B**) Hematoxylin and Eosin staining of a normal vein showing presence of cells and nuclei (blue) and cytoplasm (pink/red), (**C**) while the DV shows no staining for nuclei and the lack of a continuous endothelial layer. (**D**) Similarly, staining of a DV with Vernhoeff Von Gieson shows presence of elastin bands and collagen but no nuclei, indicating removal of cellular material. (**E**) while the normal vein shows presence of abundant nuclei (black) and elastin bands (black) and collagen (red/pink), Scanning electron micrographs of DV showing presence of abundant collagen fibers in the inner (**F**) and outer surface (**G**) of the vein. (**H**) Transmission electron micrograph showing presence of elastin bands (black staining), collagen bundles but no nuclei in the DV. Scale bars **B-E** =75µm, **F**=10µm, **F**=50µm **F**=5µm.

Figure S2: Quantification of extracellular matrix proteins and growth factors in the decellularized veins

(**A**) Quantification of extracellular matrix proteins showed no significant loss of collagen (n=4 & p=0.9) but a significant decrease in glycosaminoglycans (n=5 & p=0.041) (GAG's) in decellularized veins (DV) as compared with normals. (**B**) Quantification of various angiogenic growth factors in the normal and decellularized (DV) veins (n=2) showed the presence of (albeit low levels) several important growth factors even after removal of cells in the DV. Box and whisker diagram of work (**C**) and elongation (**D**) from tensile tests. NHV – Native human vein (n=4) DCHV - decellularized human vein (n=10) and RCHV – recellularized human vein (n=3). The DC vein segments withstand significantly lower work ($p<0.05$) compared to the corresponding native vein. The DCHV segments also showed significant lower elongation ($p<0.05$) compared to the NHV. RCHV did not significantly differ from the NHV.

Figure S3: (**A**) Negative control picture for figure 3A (**B**) Negative control picture for figure 3B - C. (**C**) Negative control picture for figure 3D – F. Immunofluorescence staining of RV with anti-CD31(**E**) and anti-alpha actin (**D**) antibodies showing presence of endothelial cell monolayer (green) on the luminal surface, smooth muscle cells (green) in the media respectively.

Figure S4: (**A**). Schematic representation of the bioreactor system showing the culture vessel containing the vein and rest of the components. The direction of arrows indicates the flow of solutions in the tubes.

Figure S1

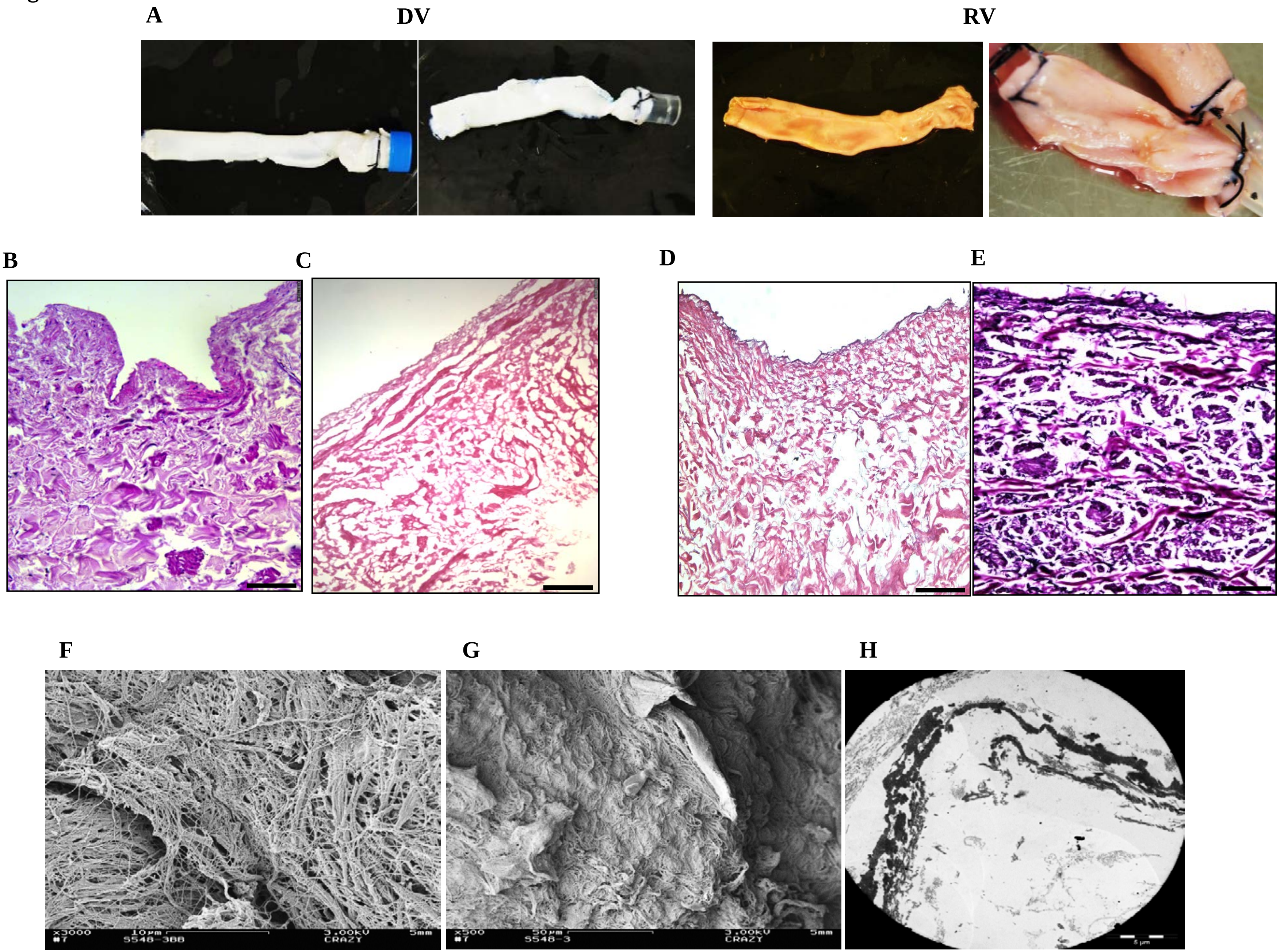

Figure S2

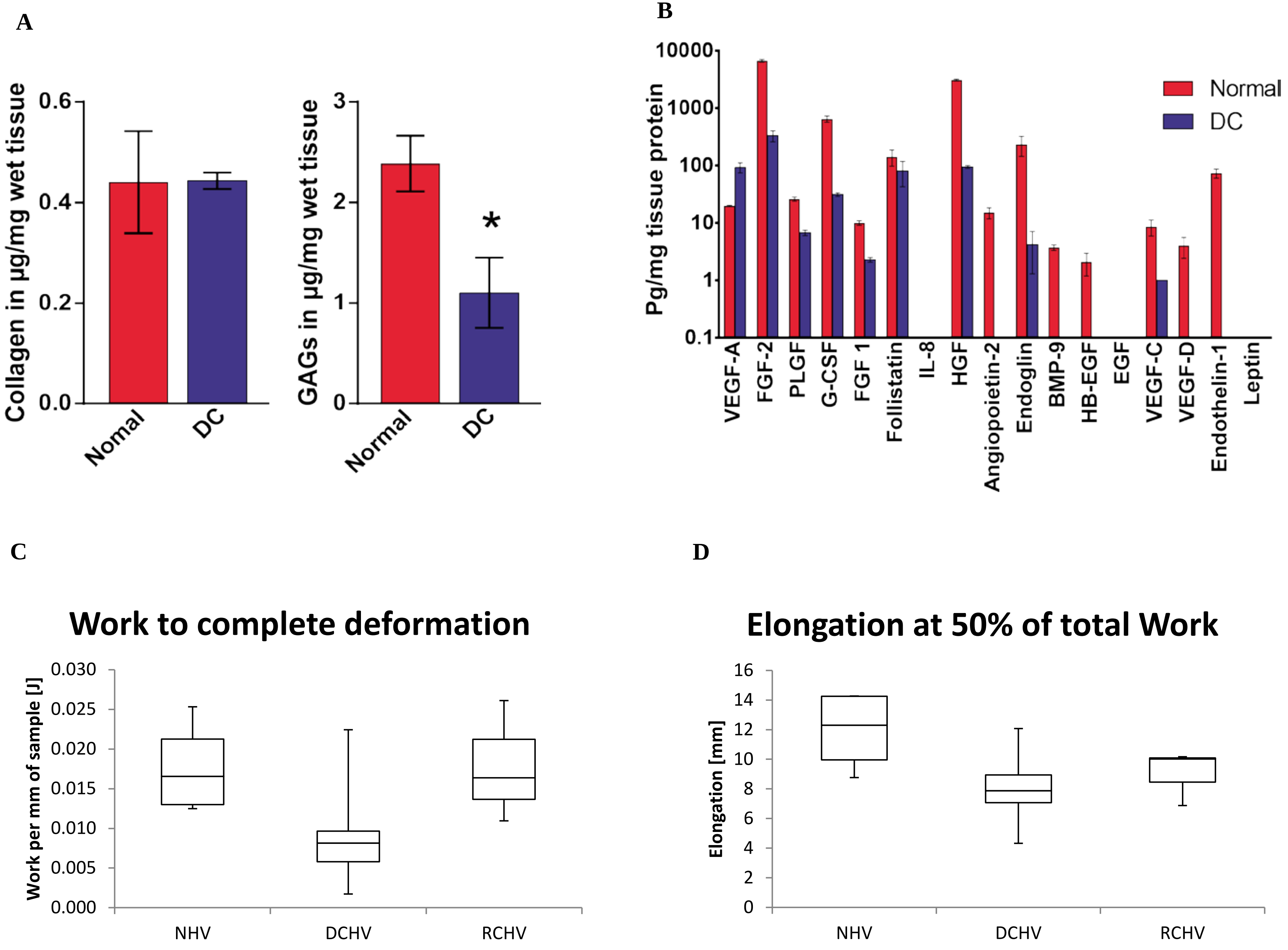
A
Collagen in µg/mg wet tissue
0.0
0.2
0.4
0.6
Nomal
DC
GAGs in µg/mg wet tissue
0
1
2
3
Normal
DC
*
B
Pg/mg tissue protein
10000
1000
100
10
1
0.1
Normal
DC
VEGF-A
FGF-2
PLGF
G-CSF
FGF 1
Follistatin
IL-8
HGF
Angiopoietin-2
Endoglin
BMP-9
HB-EGF
EGF
VEGF-C
VEGF-D
Endothelin-1
Leptin
C
Work to complete deformation
Work per mm of sample [J]
0.000
0.005
0.010
0.015
0.020
0.025
0.030
NHV
DCHV
RCHV
D
Elongation at 50% of total Work
Elongation [mm]
0
2
4
6
8
10
12
14
16
NHV
DCHV
RCHV

Figure S3

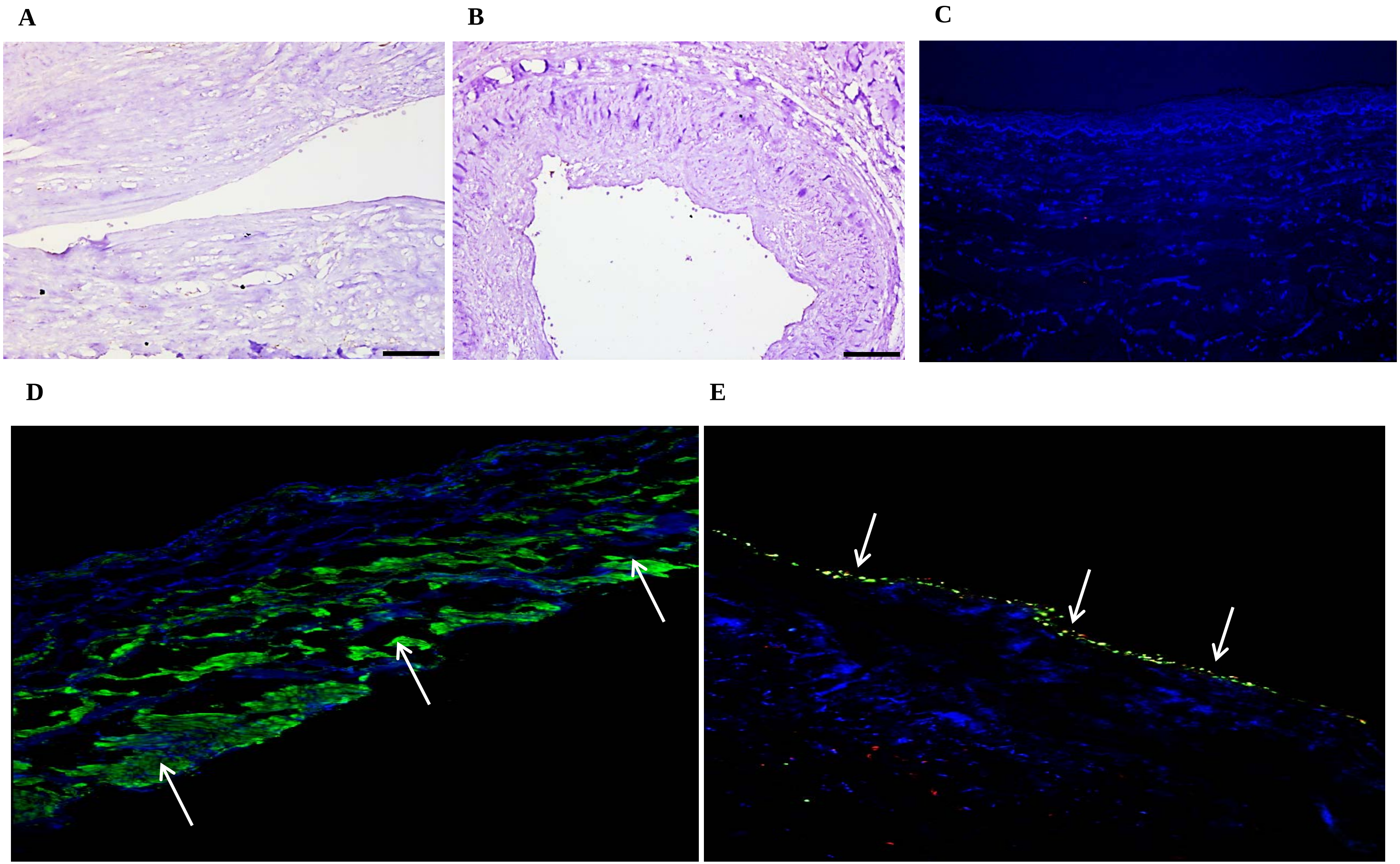

Figure S4

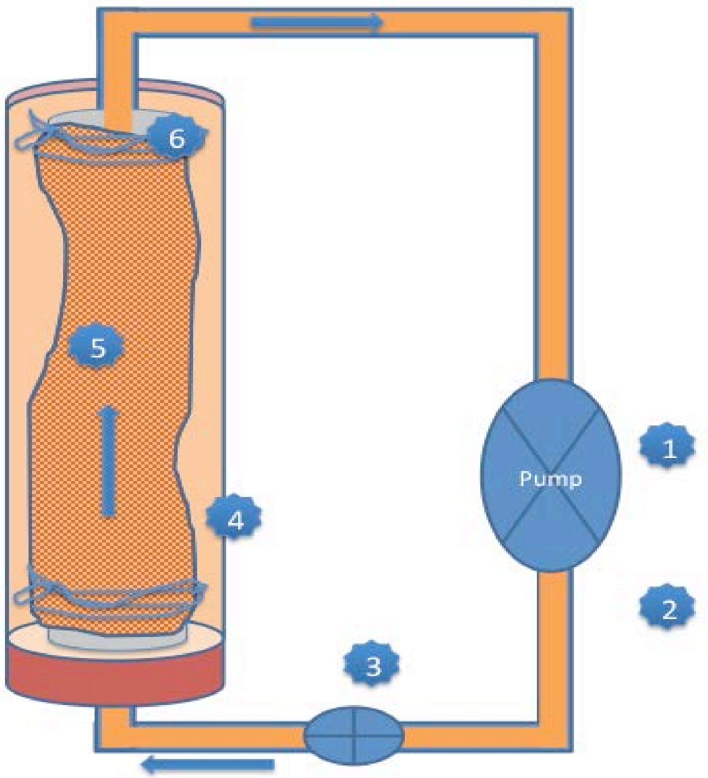

1 – Peristaltic pump
2 – 5 mm diameter silicone tubing perfusing medium in direction of arrow
3 – Bubble trapper
4 – Tissue culturing chamber
5 – Vessel scaffold
6 – Silk thread tied on adapter to secure both ends
