# Supplementary figures and images for: In Vivo Application of Tissue-Engineered Veins Using Autologous Peripheral Whole Blood: A Proof of Concept Study
Source: eBioMedicine. 2014 Sep 22;1(1):72–9. doi: 10.1016/j.ebiom.2014.09.001 (PMC4457407; doi:10.1016/j.ebiom.2014.09.001)

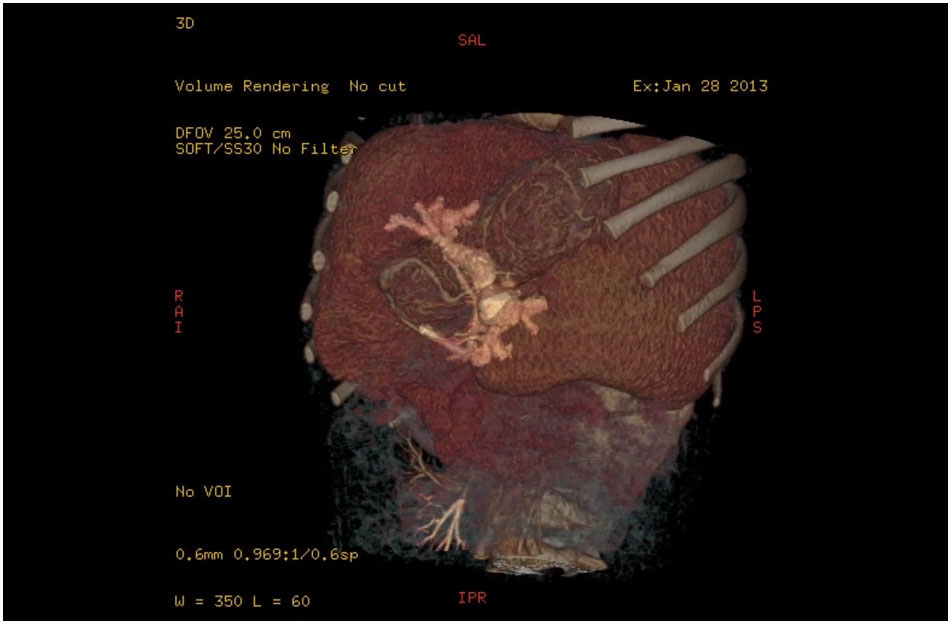

Supplement: Video — 3-D reconstructed graft. Demonstration of a good vascular bed in patient 1, 21 months post-transplantation. [file mmc2.jpg]
